# 3D Binary Mesocrystals from Anisotropic Nanoparticles

**DOI:** 10.1002/anie.202112461

**Published:** 2021-11-25

**Authors:** Christian Jenewein, Jonathan Avaro, Christian Appel, Marianne Liebi, Helmut Cölfen

**Affiliations:** ^1^ Department of Chemistry Physical Chemistry University of Konstanz Universitätsstrasse 10 Konstanz Germany; ^2^ Center for X-ray Analytics Empa—Swiss Federal Laboratories for Materials Science and Technology Lerchenfeldstrasse 5 9014 St. Gallen Switzerland; ^3^ Paul Scherrer Institut 5232 Villigen PSI Switzerland; ^4^ Department of Physics Chalmers University of Technology 41296 Gothenburg Sweden

**Keywords:** anisotropy, mesocrystal, nanoparticle, self-assembly, superlattice

## Abstract

Binary mesocrystals offer the combination of nanocrystal properties in an ordered superstructure. Here, we demonstrate the simultaneous self‐assembly of platinum and iron oxide nanocubes into micrometer‐sized 3D mesocrystals using the gas‐phase diffusion technique. By the addition of minor amounts of a secondary particle type tailored to nearly identical size, shape and surface chemistry, we were able to promote a random incorporation of foreign particles into a self‐assembling host lattice. The random distribution of the binary particle types on the surface and within its bulk has been visualized using advanced transmission and scanning electron microscopy techniques. The 20–40 μm sized binary mesocrystals have been further characterized through wide and small angle scattering techniques to reveal a long‐range ordering on the atomic scale throughout the crystal while showing clear evidence that the material consists of individual building blocks. Through careful adjustments of the crystallization parameters, we could further obtain a reverse superstructure, where incorporated particles and host lattice switch roles.

## Graphical abstract

The self‐assembly of anisotropic nanoparticles into large superstructures is a key component in the research of novel metamaterials. While the formation of mono‐phase mesocrystals has been achieved in recent years, the self‐assembly of binary structures from such particles remained highly challenging. A simple method enables the formation of binary mesocrystals from anisotropic iron oxide and platinum nanocubes in two and three dimensions.

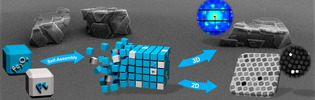

## Introduction

Nanoparticle self‐assembly is one of the most promising aspects when it comes to the generation of novel nanostructured materials.[^1^, ^2^, ^3^, ^4^, ^5^, ^6^, ^7^, ^8^] As a result, researchers have found that utilizing controlled self‐assembly can lead to astounding new materials with exciting properties and unique functionalities.[^9^, ^10^, ^11^] This is not only restricted to isotropic spherical nanoparticles, which usually self‐assemble into close‐packed superlattices, but is especially true for anisotropic nanocrystals, which have shown to achieve even more complex superstructures and directional properties.[^12^, ^13^, ^14^] Controlled self‐organization on the nanoscale has already been observed many years ago within the internal structure of BaSO_4_ or rod‐like particles of Ce^IV^ minerals.[^15^, ^16^] However, a clear and systematical description was not established until 2005 when mesocrystals were described as a new type of colloidal crystals, which formed from non‐spherical crystalline building units as oriented superstructures with common outer faces.^[17]^ Since then, the interest in mesocrystals has increased rapidly, not only due to their fascinating new characteristics combining the properties of nanocrystals with those of larger assemblies such as superparamagnetic behavior for particle sizes exceeding the range for superparamagnetism,^[18]^ but also based on their characteristic to serve as a model system for non‐classical crystallization behavior.[^19^, ^20^, ^21^, ^22^] In addition, mesocrystals received a lot of recognition due to their presence in various biological materials like nacre or sea urchin spines.[^23^, ^24^, ^25^] Based on the given IUCr definition for crystals, a more distinct definition for a mesocrystal was later proposed to be “a nanostructured material with a defined long‐range order on the atomic scale (in at least one direction), which can be inferred from the existence of an essentially sharp wide angle diffraction pattern (with sharp Bragg peaks) together with clear evidence that the material consists of individual nanoparticle building units”.^[20]^

In more recent work the controlled self‐assembly of oleic‐acid‐stabilized iron oxide nanocubes into highly ordered arrays with lateral dimensions on the micrometer scale has been conducted^[26]^ and the structure formation process was studied.[^27^, ^28^] This system was investigated in more detail to contribute knowledge crucial for the understanding of packing arrangement and orientational order of cubic iron oxide nanoparticles in two‐ and three‐dimensional superlattices.[^29^, ^30^] These findings are not exclusive to iron oxide nanoparticles, as a similar behavior could be further demonstrated on the example of platinum nanocubes.[^31^, ^32^]

When it comes to the growth of multicomponent superlattices however, the assembly process is far more sophisticated due to the distinct pairwise interactions alongside the space‐filling rules that often drive self‐assembly and potential particle fusion in the first place.[^33^, ^34^, ^35^] So‐called binary nanocrystal superlattices (BNSLs) have been investigated intensively in recent years, as the resulting metamaterials promise to show exciting new properties because of their structure‐dependent collective properties and self‐organization.[^36^, ^37^, ^38^, ^39^] Especially, anisotropic nanoparticle assemblies have been of great interest due to their shape‐dependent properties provided by an additional degree of freedom.[^40^, ^41^, ^42^, ^43^] Research, however, has also laid out that the formation of BNSLs beyond layered membranes still remains a major issue until today. To the best of our knowledge, no work was reported on binary 3D mesocrystals despite their promising potential. Herein, we report the self‐assembly of iron oxide (Fe_3_O_4_) nanocubes (IONC) as well as platinum nanocubes (PtNC) into large mesocrystalline superstructures to demonstrate for the first time the formation of binary 3D mesocrystals containing both particle types.

## Results and Discussion

**Figure 1:**
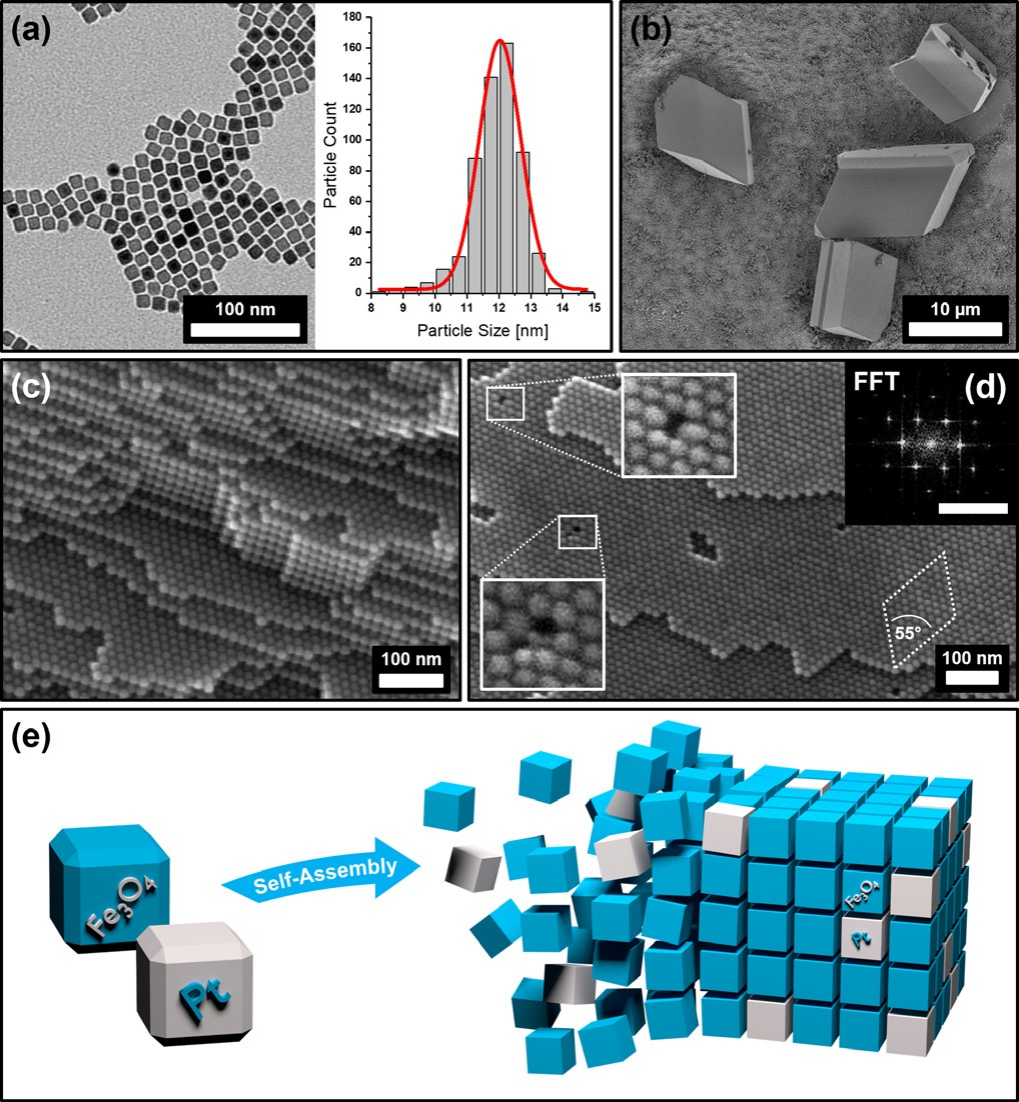
TEM image of Fe_3_O_4_ nanocubes (IONC) (a) and the size distribution of the nanoparticle dispersion (b) with an average size of 11.9±0.8 nm and a FWHM of 1.5±0.1 nm evaluated by software with the corresponding Gauss fit in red. FE‐SEM images show micrometer‐sized IONC‐based mesocrystals with distinct tetragonal habit (b) as well as their bulk (c) and surface (d) ordering of the nanoparticles. FFT analysis of the mesocrystal surface (insert, scale bar 0.2 nm^−1^) reveals a tetragonal particle ordering. Magnification of the point defects displays the incorporation of “misfit” particles within the otherwise perfect superlattice. Figure (e) illustrates the concept of a random platinum nanocube (PtNC, gray) incorporation into a mesocrystalline IONC (blue) host lattice.

IONCs were synthesized using a two‐step process, first synthesizing an iron oleate precursor to subsequently form ca. 12 nm sized, monodisperse cubes stabilized by oleic acid in various organic solvents, such as toluene, hexane or tetrahydrofurane (Supplementary Information, Materials and Methods). The resulting particles were then recrystallized in order to obtain a stable 11.9±0.8 nm IONC particle dispersion with a much narrower size distribution by utilizing the gas‐phase diffusion method to form large mesocrystals on a silicon substrate prior to redispersing them in the preferred solvent (Supplementary Information 1). This method has already been reported for cubic iron oxide as well as platinum nanoparticles.[^32^, ^44^] A recrystallized particle sample is shown in Figure 1 a. In order to gain insight into the specific mesocrystal structure of our samples with regards to applicable characteristics, which can be exploited for a binary mesocrystal formation, further in‐depth experiments were conducted. Recrystallized IONCs have been assembled from hexane into tetragonal‐shaped 10–30 μm sized mesocrystals by using the gas‐phase diffusion technique (Figure 1 b) and subsequently broken apart with a micromanipulator to reveal their inner bulk structure. Field‐Emission Scanning Electron Microscopy (FE‐SEM) imaging at very high resolutions displayed that our IONC particles preferably self‐assemble into stacked layers of hexagonally ordered particles (Figure 1 b–d). Fast Fourier Transformation (FFT) of the particle arrays (Figure 1 d, insert) displays a *c*2*mm* symmetry due to the slight distortion of the hexagonal ordering as a result of the “bump‐to‐hollow” packing principle of IONCs.^[29]^ These findings suggest a tetragonal crystal system, which is in good agreement with their mesocrystal habit as seen in Figure 1 b.

In addition, the FE‐SEM images reveal interesting features of the inherent defects within the mesocrystalline superlattice as illustrated in Figure 1 d. The most common imperfections of these mesocrystals are point defects, which often incorporate differently sized or shaped particles into the vacancy of the predominant particle lattice. Although mesocrystals show similarities when compared to a classical atomic crystal in many ways, one key difference can be found in their inherent building block uniformity. Whereas atoms always exhibit the exact same size and shape, nanoparticle dispersions in contrast possess a wide range of these attributes, no matter how narrow their size distribution is. We suppose that small amounts of “misfit” particles are implemented during crystal growth as a result of defect formation due to the slight polydispersity of the parent particle dispersion. These findings further suggest that the formation of our mesocrystals is not exclusively limited to particles of perfect size and shape in contrast to the formation of atomic crystals. They further imply a strong correlation between the composition of the parent particle dispersion and their resulting mesocrystalline structure. We conclude that the incorporation of particles of different material (such as platinum) into a host system of highly ordered nanocube arrays may be accessible by exploiting this behavior. However, it is still very important to utilize nanoparticles of a similar size and shape in order to promote the probability of foreign particle incorporation within the host lattice as the illustration of a binary superlattice in Figure 1 e implies. To test our assumptions, we synthesized PtNCs using oleic acid as stabilizing agent to retrieve stable particle dispersions in various organic solvents, particularly in toluene and hexane with a similar size and shape (Supplementary Information, Materials and Methods). Based on the obtained particle size of the PtNCs, the previously described two‐step synthesis of IONCs had to be tuned to achieve a complementary particle size by increasing the heating ramp rate and reducing the duration at reflux temperature as further annotated in the Supplementary Information 1.

**Figure 2:**
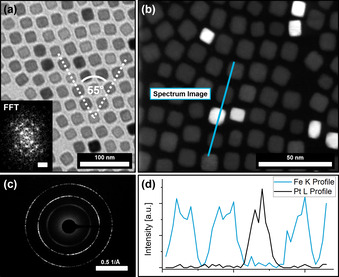
(a) shows a TEM image of a 2D binary self‐assembly of a PtNC/IONC particle mixture from hexane with the corresponding FFT (insert) indicating a hexagonal particle ordering of *c*2*mm* symmetry. HRTEM and HAADF analysis (b) show the random incorporation of PtNC into the IONC lattice. Complementary SAED (c) displays a preferred particle orientation on the atomic scale. An EDX line scan (d) of the binary particle assembly further confirms that the high contrast particles are PtNCs.

The so tailored nanocubes of platinum (11.5±1.1 nm) and iron oxide (11.9±0.8 nm), likewise stabilized by oleic acid in hexane, differ less than 0.5 nm in their size average according to a software‐based particle analysis (Figure 1 a) and were therefore utilized to conduct all further investigations into the binary self‐assemblies. First, the fabrication of 2D binary monolayers was conducted by self‐assembling a stable particle dispersion of an IONC and PtNC mixture with a 20 to 1 (IONC to PtNC) mass ratio over the course of 60 minutes via the solvent evaporation technique. A strong excess of IONCs was chosen to facilitate the incorporation of PtNCs within the IONP host lattice. In order to keep the evaporation speed at a low rate, the solvent was evaporated in a partially saturated atmosphere of hexane. The resulting arrays of the binary particle mixture were characterized by Transmission Electron Microscopy (TEM) techniques as shown in Figure 2.

Figure 2 a indicates an even distribution of PtNCs within IONC arrays roughly matching the initial mixing ratio of 20 to 1 (IONC to PtNC) as revealed by statistical analysis that determined a PtNC content of 4.35 % (Supplementary Information 3). The high atomic number of platinum atoms leads to a stronger contrast in the TEM images revealing the positions of the PtNCs within the assembly. Evaluation of the observable lattice planes of each particle in High‐Resolution TEM images consolidate this presumption (Supplementary Information 2). To confirm this, we conducted High‐Resolution Scanning Transmission Electron Microscopy (HR‐STEM) to map the particle arrays with a High Annular Dark‐Field Detector (HAADF), which detects the incoherently scattered electrons from the atomic nuclei and is therefore highly sensitive to the variations in the nuclear charge number of our material. Figure 2 b shows such a map, where the PtNCs can clearly be distinguished from the IONCs. Additionally, an Energy Dispersive X‐Ray (EDX) line scan along such a binary particle array was performed as shown in Figure 2 b,d to confirm our observation. In general, the PtNCs were found to be randomly distributed inside the host lattice, replacing single IONCs at their positions. Although somewhat distorted, the hexagonal particle ordering of the IONC assemblies can still be observed regularly within the examined particle assembly domains in contrast to the more cubic ordering which is usually preferred by the PtNCs (Figure 2 a, insert). The overall structure, however, appears to be only slightly less ordered when compared with pure IONC assemblies. These findings affirm our presumptions that the prepared particle mixtures can self‐assemble into highly ordered binary arrays while retaining the superstructure provided by the host lattice of the abundant particle type. By utilizing Selected Area Electron Diffraction (SAED) it can further be observed that the particles assemble in a mesocrystalline manner as a preferred particle orientation on the atomic scale is indicated by the electron diffraction pattern in Figure 2 c.

We further found that the aforementioned discoveries can be applied to obtain three‐dimensional self‐assemblies from the same IONC/PtNC particle mixtures (20:1 mass ratio) by utilizing the gas‐phase diffusion technique. The superlattice based on the structure of IONC‐based mesocrystals was once more chosen to provide the host network in which PtNCs are randomly incorporated by replacing IONCs on their positions during the mesocrystal formation. Analogous to our findings in the two‐dimensional binary particle arrays we used mixtures with a particle ratio of approximately 10 to 1 (IONC to PtNC) in regards to IONC surplus.

**Figure 3:**
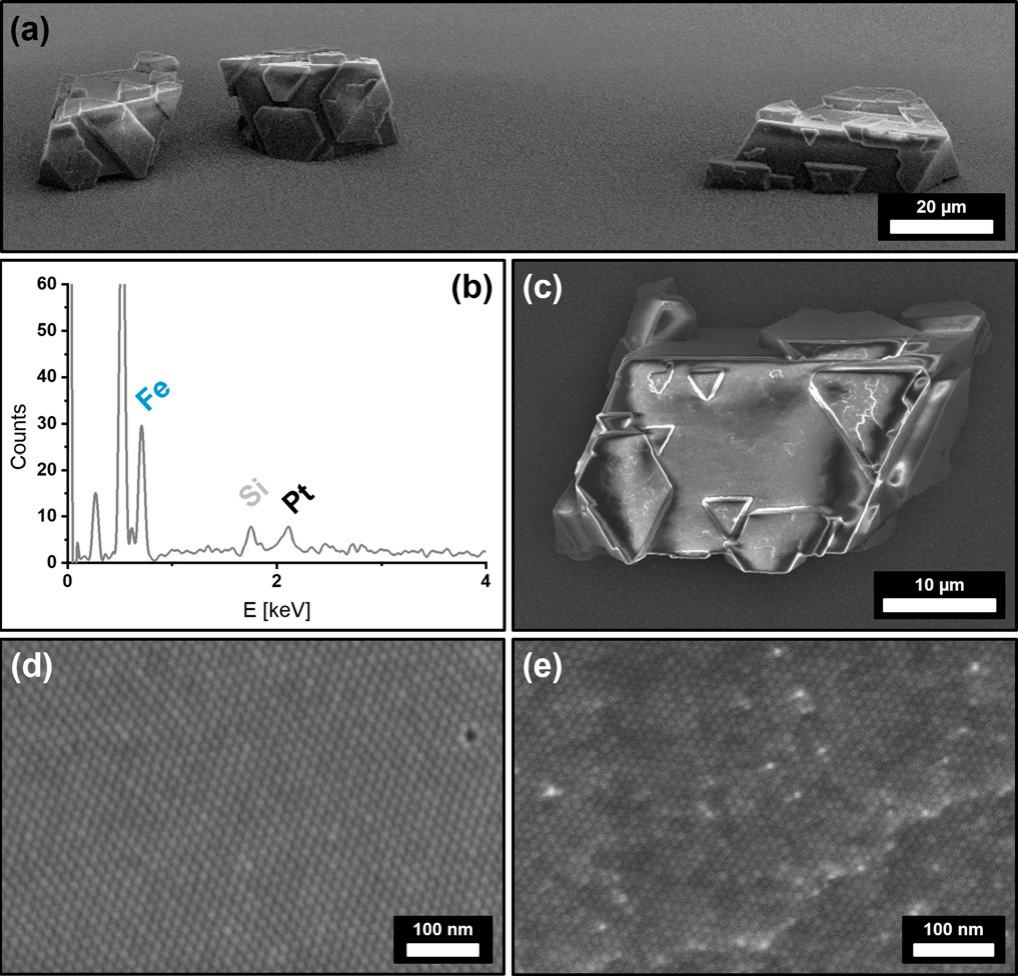
FE‐SEM images of IONC/PtNC binary superstructures from hexane (a,c,e) at different magnifications. Image (a) shows the distinct crystal shape of the obtained superstructures. Complementary EDX analysis (b) of a binary crystal (c) reveals a significant amount of Pt incorporated within a predominantly Fe‐based superstructure. High‐resolution FE‐SEM images of the superstructure surfaces show the comparison of a pure IONC mesocrystal (d) with a binary superstructure (e). High‐contrast particles indicate the presence of randomly incorporated PtNCs into a IONC superlattice.

Extensive and highly ordered superstructures with distinct crystal shapes formed from hexane over the course of 7 days as shown in Figure 3 a. The obtained mesocrystals are 20–40 μm in size and emerge with a distinct crystal shape, accompanied with a multitude of crystal twinning on their outer perimeter (Figure 3 a,c). To confirm the binary composition of the superstructures, we conducted EDX scans in order to determine their elemental composition. A significant amount of platinum within the scanned crystals was found as shown in Figure 3 b. The observable geometry of the crystals further suggests that the general structure of the host lattice remains mostly unchanged by the incorporation of foreign platinum nanocubes in comparison to the previously observed crystal habit of analogous mesocrystals from pure IONC dispersions in Figure 1 b. The predominant increase in crystal twinning, however, proposes a higher rate of defect formation during crystal growth as they are mostly absent within the pure IONC‐based superstructures. We assume that this is most likely a result of the incorporation of platinum particles into the iron oxide superlattice. While high‐resolution FE‐SEM imaging illustrates the hexagonal ordering of the nanoparticle arrays it also implies the indicated platinum nanoparticle incorporation. As shown in Figure 3 e, randomly distributed particles with a significant difference in contrast in‐between neighboring particles can be seen. This finding cannot be observed for pure mesocrystal surfaces as the comparison of a IONC‐based mesocrystal surface in Figure 3 d shows. As materials with a high atomic number exhibit a much stronger contrast in FE‐SEM imaging, the presence of randomly incorporated PtNCs within highly ordered IONC arrays is highly suggested. In regards to the performed EDX analysis in Figure 3 b we further conclude that the PtNCs are likewise not only a surface anomaly but rather reflect the actual bulk composition of the crystals given the penetration depth of a 10k eV electron beam of several hundred nanometers.

**Figure 4:**
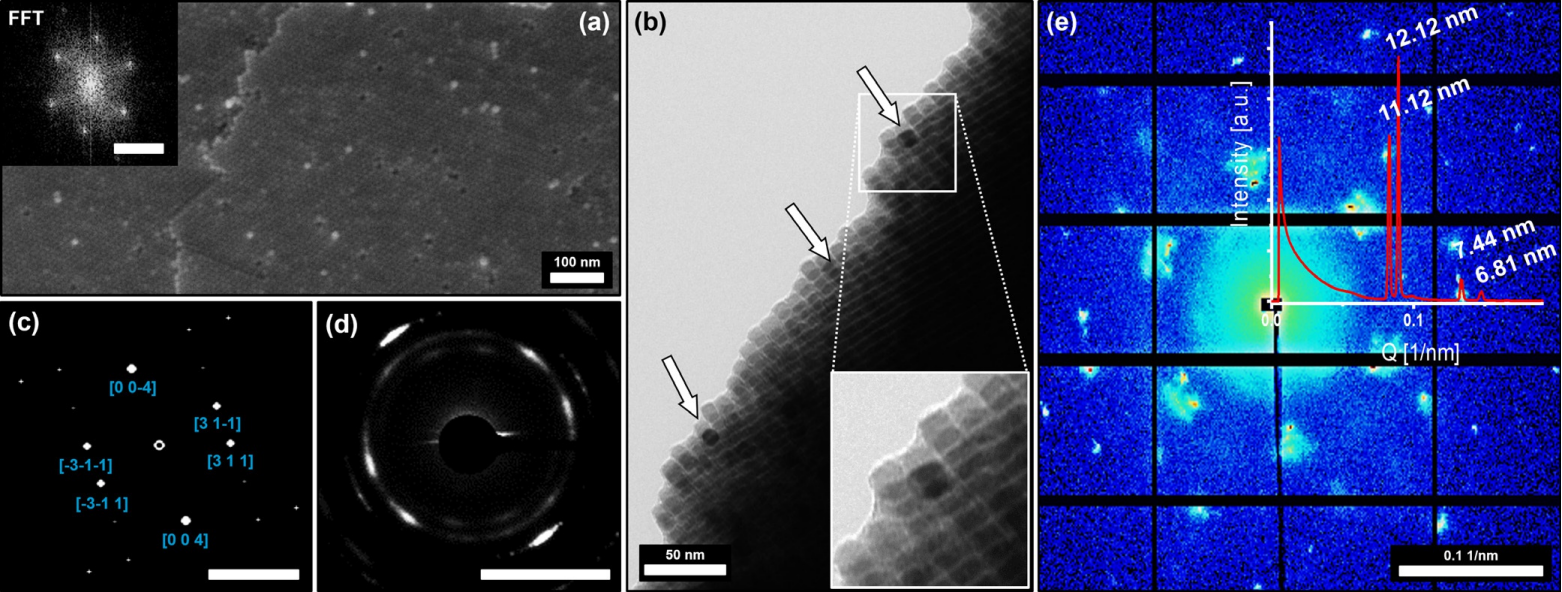
High‐resolution FE‐SEM and TEM images of a broken crystal piece revealing the binary superstructure's inner surface (a) and its bulk (b) indicating a hexagonal particle ordering further confirmed by a FFT (insert a). The TEM imaging performed alongside the edge of the broken binary crystal (b) illustrates the incorporation of high‐contrast PtNCs within the bulk of the IONC superlattice (indicated by arrows). SAED performed on the thin edge (d) further reveals the atomic ordering of the Fe_3_O_4_ lattices as viewed from a [130] zone axis, which is confirmed by the simulated diffraction pattern shown in (c) proving the mesocrystal nature of the binary superstructures. Synchrotron‐based SAXS analytics performed on a single binary superlattice crystal (e) shows a distinct packing structure indicated by the symmetry of the shown reflexes. Shape and position of the signals further allow the determination of particle distances (12.1 nm and 11.1 nm) and gaps (2.5 nm) as discussed in more detail in the Supplementary Information 5. Scale bar in (a) insert is 0.1 nm^−1^ and bars in (c) and (d) are 5 nm^−1^.

A more detailed investigation of the bulk structure, however, proves to be challenging as Focused Ion Beam (FIC) cutting techniques have to be disregarded due to the high energies introduced at the cutting plane resulting in a significant altering of the nanoparticle shape and ordering. We therefore utilized micromanipulators in order to fracture the crystals, which allows additional FE‐SEM imaging of their inner structure. In good agreement with the observations on the crystal surface, high‐contrast particles as well as a high amount of defects within the particle lattice bulk can be observed in Figure 4. Both EDX and FE‐SEM analytical methods were furthermore used to perform a statistical analysis of the PtNC content within the IONC host lattice (Supplementary Information 3). PtNC particle detection on the broken crystal interface gives a 0.61 % PtNC ratio, which is in good agreement with a quantitative EDX evaluation showing a 0.68±0.04 % PtNC/IONC ratio. In comparison to the 4.35 % PtNC content for 2D binary assemblies, it can be stated that the incorporation of particles occurs at a much lower rate for 3D binary self‐assemblies through the gas‐phase diffusion technique than it does for 2D binary monolayers through solvent evaporation. A detailed FFT of the FE‐SEM imaged superlattice in Figure 4 a confirms the already observed hexagonal packing of the binary nanocube mixture in one plane whereas the ordering of the bulk (Figure 4 b) further displays a cubic stacking of the planes analogous to a pure IONC‐based mesocrystal as shown in Figure 1 c. This suggests that the incorporation of PtNCs does not significantly affect the particle arrangement of the IONCs as its structure is almost identical.

Characterization of the superlattice packing remains very challenging and can be difficult to be conducted without the help of a focused X‐ray beam within the size range of the crystal size. Therefore, synchrotron‐based SAXS measurements of a single binary mesocrystal were performed at the Swiss Light Source cSAXS beamline at the Paul Scherrer Institute with a micro‐focused monochromatic synchrotron‐based X‐ray beam of 27×16 μm revealing the particle ordering of the binary crystals in unprecedented detail. Two strong sharp signals at 0.0825 nm^−1^ and 0.0894 nm^−1^ are in good accordance with the estimated particle size of 11.5±1.1 nm. Further reflexes at 0.1339 nm^−1^ and 0.1476 nm^−1^ paired with the symmetry of the diffraction pattern as shown in Figure 4 e suggest a body‐centered tetragonal (BCT) packing of the particles. The provided SAXS pattern is in good agreement with a [111] zone axis for a unit cell with the determined parameters of *a=b=*34.24 nm, *c=*29.24 nm and an *I*4/*mmm* space group as displayed in Supplementary Information 4. Resolution of the exact crystallographic structure however would require further verification by additional synchrotron SAXS measurements at various angles. These results approve the observed crystalline structure as previously indicated by FE‐SEM imaging and show the presence of an average inter‐particles gap value over all directions of the hexagonal packing of ca. 2.5 nm via the single analysis of 2D segmented scattering pattern (Supplementary Information 5).

**Figure 5:**
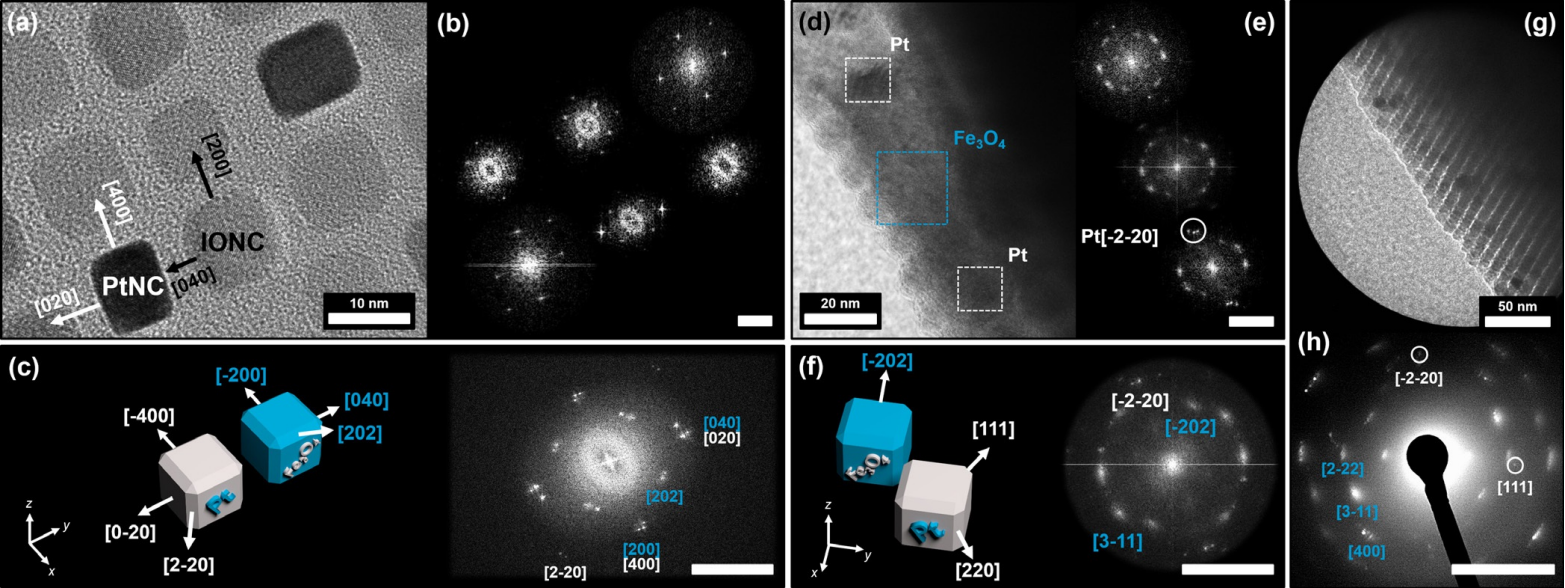
High‐resolution TEM images of binary self‐assemblies and their corresponding evaluated FFT patterns. Image (a) displays a mesocrystalline PtNC/IONC binary monolayer. The high‐contrast (darker) particles can be identified as PtNCs by the means of selected area FFT for each particle as shown in the corresponding FFT map in image (b) and Supplementary Information 2. Evaluation of the FFT patterns allows the determination of the [001] zone axis as well as the individual particle orientation as illustrated by the combined FFT in image (c) underlining a face‐to‐face orientation of PtNC to IONC as already indicated by the HRTEM image in (a). Images (d) and (e) show a similar identification for a 3D PtNC/IONC binary mesocrystal alongside a thin broken crystal piece as illustrated earlier. Although a clear zone axis cannot be determined and stacked particles give a mixed selected area FFT (e) for the high‐contrast particles, it is still evident for PtNC due to the characteristic [220] lattice parameters which are referenced in illustration (f) for the combined FFT. Because of the angles between the observable FFT signals in (f) and the reflex pattern obtained by a SAED (g and h) it can be stated that the orientation of PtNCs and IONCs within the mesocrystals is parallel as they share the same crystallographic coordinates. In all FFT and ED images Pt reflexes are indicated in white, Fe_3_O_4_ reflexes are indicated in blue and the scale bar is 5 nm^−1^.

The previously described fragmentation of the crystals also enabled TEM imaging alongside the edges of broken crystal pieces, displaying the presence of high‐contrast platinum particles embedded within an IONC matrix. Complementary SAED measurements also show a preferred direction of the wide‐angle diffracted electrons in form of high intensity arcs (Figure 4 d), which are directly correlated to the orientation of the particles. Given the crystallographic parameters of a Fe_3_O_4_ magnetite unit cell, the [131] zone axis is correlated to the highest intensity reflexes in the SAED (Supplementary Information 4). This shows that the nanoparticles not only arrange within a highly ordered superlattice, they also orient themselves along their crystallographic planes. The combination of both measurements proves that our superstructures are a nanostructured material which consists of individual nanoparticles with a defined long‐range order on the atomic scale in at least one direction. Based on these results, we further specified the observed binary superlattices to be binary mesocrystals rather than colloidal crystals that do not necessarily have the unique feature of a long‐range particle orientation based on their atomic lattice planes. In addition, we investigated the orientation of the incorporated PtNC with this method as illustrated in Figure 5. Based on HRTEM images of a binary PtNC/IONC monolayer (Figure 5 a) it is evident that both particle types orient themselves face‐to‐face alongside the same crystallographic reference frame. This is highlighted by a combined FFT over multiple particles in Figure 5 c which shows an alignment of lattice parameters for the equivalent miller indices with only minor deviations as a result of slight particle misalignment, which is the primary reason for the diffraction arcs mentioned earlier. Identical observations could be conducted alongside the broken crystal edges for 3D binary mesocrystals from the same particle mixtures. Although the HRTEM images give a mixed FFT pattern due to the stacking of multiple particles, a clear localization of high‐contrast platinum particles is furthermore possible as demonstrated in Figure 5 d–f. Distinct platinum signals corresponding to the [220] lattice plane visible by the means of FFT, in correlation to the Fe_3_O_4_ signals, allow a determination of the particle orientation within a crystallographic reference frame regardless of a provided zone axis as illustrated in Figure 5 f. In combination with SAED analysis (Figure 5 g,h) we were therefore able to identify the orientation of the PtNCs within the IONC host lattice to be parallel to each other as they share the same crystallographic reference frame.

Complementary to the formation of IONC‐based mesocrystals, which served as the primary host lattice for the binary mesocrystals in this work, we reported on the formation of pure PtNC‐based mesocrystals. It was therefore standing to reason to investigate the possibility of the formation of a “reverse” binary mesocrystal by incorporating IONCs into a PtNC host lattice. The experiments were conducted using the same particles and solvents but inverted particle concentration ratios. We found that the formation of PtNC host lattice binary particle assemblies cannot be obtained at the higher concentration levels at which the IONC host lattice binary mesocrystals were formed. The concentration of the particle mixture had to be significantly reduced by a factor of 5, to avoid mono‐phase formation of pure IONC‐ and pure PtNC‐based mesocrystals separately. We hypothesize here that the reason behind the impossibility to form reverse binary mesocrystals at high particle concentrations lies in the formation kinetic discrepancy between PtNC and IONC as reported in our preceding research.[^32^, ^44^] While the formation of a PtNC host lattice takes 10–14 days in hexane, the development of IONC‐based mesocrystals usually occurs in less than 7 days. This results in the consumption of the majority of the IONCs before the PtNC host lattice can form. We assume that a reduced particle concentration prevents the IONCs from reaching a critical particle concentration (CPC) during the gas‐phase diffusion process within the set crystallization period of 14 days, which would otherwise trigger IONC cluster formation and ultimately result in a mono‐phase mesocrystal formation.[^19^, ^45^]

**Figure 6:**
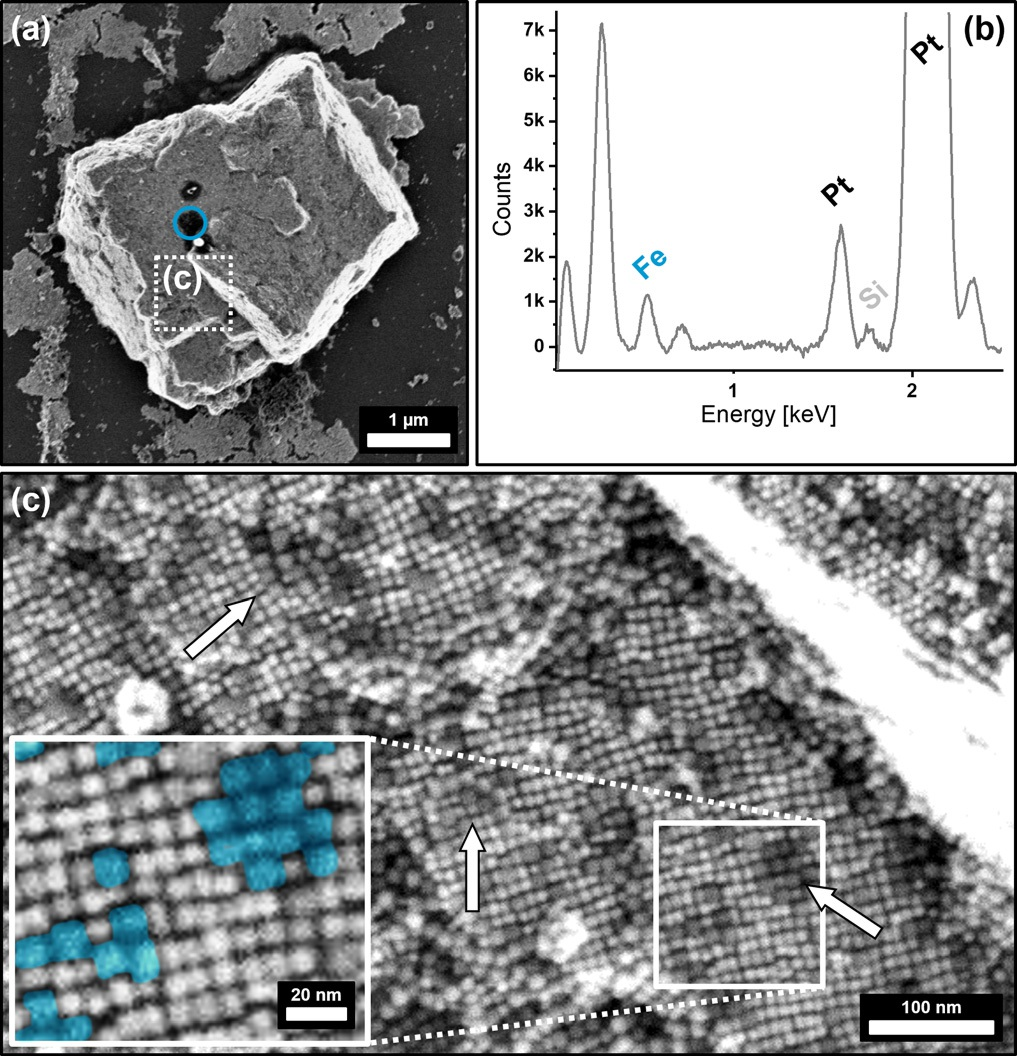
FE‐SEM images (a,c) and the corresponding EDX point analysis (b) of an inverted PtNC/IONC binary superlattice where PtNCs provide the host lattice of the superstructure. The EDX spectrum in (b) is taken from the region highlighted in blue (a) and shows a predominant amount of Pt within the crystal, accompanied with a Fe peak indicating the successful incorporation of IONCs. High‐resolution SEM of the superstructure surface in (c) further displays a binary composition of the superlattice with a random incorporation of low‐contrast (darker) particle clusters, presumably IONCs, which are indicated by arrows and highlighted at higher magnification colored in blue within the provided insert.

Nevertheless, the self‐assembly of a less concentrated binary particle mixture resulted in the formation of 3–5 μm sized, uniform superlattices, which are much smaller in size than the compared IONP host lattice binary mesocrystals. As previous research has shown that the obtained mesocrystal size is directly connected to the concentration of the parent particle dispersion,^[45]^ our results confirm that such assumption is also valid for the formation of binary mesocrystals.^[46]^ Besides the smaller size of the crystals, FE‐SEM images shown in Figure 6 a,c display an increase in IONC incorporation when compared to the respective PtNC incorporation of an IONC host lattice binary mesocrystal. Complementary EDX analysis confirms the observed prevalence of IONC within the PtNC superlattice as demonstrated in Figure 6 b. The difference between the IONCs and the PtNCs is once again indicated by the brightness of the particles, as the darker (less contrast), slightly larger IONCs are embedded into the cubic ordering of the PtNC host lattice as illustrated in Figure 6 c. The cubic ordering of the particles further reinforces our assumption that the PtNCs provide a host network of particles in which the IONCs are incorporated. It is worth noting that the IONCs preferably form small clusters of several particles, which are evenly spread inside the superlattice but do not develop into larger domains.

## Conclusion

We were able to define the optimal particle size, shape, concentration and stabilizer for a predominant host lattice mesocrystal structure to incorporate tailored particles of another material. Ultimately, the formation of highly ordered binary mesocrystals from anisotropic nanoparticles in two or three dimensions depending on the synthesis method—solvent evaporation and slow gas‐phase diffusion, respectively—could be demonstrated.

Furthermore, our observations reveal the importance of individual particle concentrations as a critical role in the development of a binary mesocrystal. We herein propose the concept of a critical particle concentration (CPC) as the driving force towards individual mono‐phase mesocrystal formation at different crystallization times. It appears thus essential that the concentration of the particle type undergoing incorporation into a host lattice must not exceed its CPC otherwise it will lead to a mono‐phase mesocrystal formation considerably earlier than by the host lattice particles hindering the formation of a binary superstructure.

While our extended experiments clearly demonstrate the feasibility of the formation of an inverse binary superlattice, they also illustrate that a variety of factors may play a crucial role in their formation as indicated by the presence of particle clusters and the overall mesocrystal habit. As a result, multiple open questions remain, such as the influence of the particle mixing ratio on the binary mesocrystal structure, the transition regime between tetragonal and cubic packing and the possibility to obtain binary and even ternary mesocrystals just to name a few. These topics need to be addressed in future work, but it is already clear that 3D binary mesocrystals open up a completely new range of possible complex multicomponent superstructures with new features, packing orders as well as physical and chemical properties that are yet to be discovered.

## Conflict of interest

The authors declare no conflict of interest.

## Supporting information

As a service to our authors and readers, this journal provides supporting information supplied by the authors. Such materials are peer reviewed and may be re‐organized for online delivery, but are not copy‐edited or typeset. Technical support issues arising from supporting information (other than missing files) should be addressed to the authors.

Supporting Information
