# Supplementary material for: 3D Binary Mesocrystals from Anisotropic Nanoparticles
Source: Angew Chem Int Ed Engl. 2021 Nov 25;61(2):e202112461. doi: 10.1002/anie.202112461 (PMC9298807; doi:10.1002/anie.202112461)
Supplement: Supplementary file 1 — Supporting Information [file ANIE-61-0-s001.pdf]

Angewandte Chemie
Eine Zeitschrift der Gesellschaft Deutscher Chemiker

Supporting Information

3D Binary Mesocrystals from Anisotropic Nanoparticles

*Christian Jenewein, Jonathan Avaro, Christian Appel, Marianne Liebi, and Helmut Cölfen**

anie_202112461_sm_miscellaneous_information.pdf

Supporting Information

Materials and Methods

Chemicals

Iron(III) chloride hexahydrate from Sigma-Aldrich, magnesium sulfate hydrate (>99%) from Carl Roth and sodium oleate (>97%) from TCI were used for the iron oxide nanocube synthesis. Tungsten hexacarbonyl (99%) and platinum(II) acetylacetonate (98%) for the platinum nanocube synthesis were purchased from abcr. Linoleic acid (99 %), toluene (99,8+%) and oleylamine with a C-18 content of 80-90% were provided by Acros Organics. Oleic acid (99%) was received from Alpha Aesar. Tetrahydrofuran (100%), hexane (98%) and ethanol (99,8+%) were purchased by VWR and Roth respectively. All chemicals were used without further purification.

Iron (III) Oleate Precursor Synthesis

Into a 100 ml two-neck Schlenk flask was added Iron (III) chloride hexahydrate (1.35 g, 5 mmol) and sodium oleate (4.58 g, 15 mmol) under nitrogen atmosphere, then degassed and subsequently dissolved in a mixture of ethanol (10 mL), hexane (18 mL), and MilliQ water (8 mL). The mixture was then frozen in a liquid nitrogen bath in order to Freeze-Pump-Thaw it for one cycle. The reaction mixture was then heated to 80°C while stirring and refluxed for four hours. The resulting organic phase was washed with milliQ water (3 x 50 mL) and dried over $Mg(SO_4)_2$ to remove water residues. Afterwards, rotary evaporation at 50 °C / 330 mbar was used to remove organic solvents, leaving a dark brown oil as crude product which is stored at 4 °C until further use.

Iron Oxide Nanocubes Synthesis

The heating-up method was utilized to form iron oxide nanocubes from the Iron (III) Oleate Precursor. The previously prepared iron (III) oleate (4.54 g, 5 mmol), as well as sodium oleate (218 mg, 0.72 mmol) and 99% pure oleic acid (227 µL, 0.72 mmol) were solved in 1-octadecene (25 mL) within a three-neck flask and dried under vacuum at 60 °C for 30 min while stirring to remove remaining water. The reaction mixture was then heated to reflux temperature using a heating ramp of 3.3 °C/min and stirred for further 30min at this temperature before cooling back to room temperature. The resulting black liquid was transferred into falcon tubes and purified via centrifugation for several times with double the amount ethanol at first (1500 rpm for 15 min, two times) and then with a mixture of ethanol to toluene decreasing each washing cycle (4:1, 3:1, 2:1) for three times at 9000 rpm (15 min). In addition, larger agglomerates can be filtered after dispersing the highly viscous black product in various organic solvents such as THF, toluene or hexane.

Platinum Nanocubes Synthesis

Platinum nanocubes have been synthesized according to a slightly modified procedure, which has already been reported by Zhang et al.[1] A mixture of 40 mg platinum(II) acetylacetonate and 3.56 g of oleic acid in 16 ml of oleylamine was placed in a two-neck Schlenk flask equipped with a condenser and attached to a Schlenk line. While vigorous stirring, the mixture was heated to 120 °C under Nitrogen atmosphere. After the addition of 100 mg of

tungsten hexacarbonyl, the solution was raised to 240 °C over the course of 45 minutes. At this temperature, the solution was continuously stirred for further 45 minutes under weak Nitrogen flow before cooling to room temperature. The crude product was separated by centrifugation at 9000 rpm for 20 minutes and washed with anhydrous hexane before precipitating with ethanol in three cycles. A black and oily solid was obtained as the final product and dispersed in either hexane, toluene or tetrahydrofuran and stored under light exclusion until further use.

Mesocrystal Formation via Gas-Phase Diffusion

Into a 1 ml flat bottom glass, 300 µL of a prepared particle dispersion containing 3 µl/ml oleic acid (99%) and a cleaned 5x7 mm double side polished silicon wafer snippet were added. The silicon snippet was cleaned by gradual ultrasonification in ethanol, iso-propanol, acetone, ethylacetate, toluene and toluene p.a. for 10-15min each. The prepared 1 ml flat bottom glass was then placed into a 5 ml screw cap vial containing 1.5 ml of an ethanol/dispersion solvent (50:50) mixture. The vial was then stored in a desiccator containing an ethanol rich atmosphere for several days, depending on the dispersion solvent (THF: 1-2 days, Toluene: 7-14 days, Hexane: 14+ days). After crystallization, the silicon snippet was carefully removed, immersed into pure ethanol for five seconds and dried on air. Particle dispersion mixtures for binary self-assemblies were used at the following concentrations:

- IONC host lattice PtNC/IONC binary mesocrystals – 100 µL of a 1 mg/mL PtNC dispersion in hexane and 200 µL of a 10 mg/ml IONC dispersion in hexane.
- PtNC host lattice IONC/PtNC binary superlattices – 275 µL of a 1 mg/mL PtNC dispersion in hexane and 25 µL of a 10 mg/ml IONC dispersion in hexane.

Self-Assembly formation via Solvent Evaporation

A 15 µL drop of a stable particle dispersion mixture containing 3 µL/ml additional oleic acid (99%) was carefully placed on a substrate and slowly evaporated within a partially saturated atmosphere of the dispersion solvent within a desiccator. After evaporation, the residual oleic acid can be removed by plasma cleaning for 2 minutes prior to FE-SEM analysis.

Analytical Instruments

Transmission Electron Microscopy (TEM) analysis was performed on a ZEIS LIBRA120 instrument using 200 mesh carbon coated copper grids equipped with a 120 kV Lanthanum hexaboride emitter and a Koehler illumination system. High resolution TEM imaging was conducted on a JEOL JEM-2200FS equipped with ZrO/W(100) Schottky field emission gun (operated at 200 kV), an in-column Omega-type filter as well as a STEM Bright Field (BF) and High-Angle Annular Dark-Field (HAADF) detector. FE-SEM imaging was carried out on a Carl Zeiss Gemini 500 Microscope equipped with multiple detectors for secondary and backscattered electrons using acceleration voltages of up to 10 kV. Samples were plasma cleaned for 60-120 seconds using a MiniFlecto from Plasma technologies for better imaging. To perform Energy Dispersive X-Ray (EDX) measurements, the microscope is also equipped with an EDX Ultim Max 100 Silicon Drift Detector from Oxford Instruments. PXRD measurements were conducted using a Bruker D8-Discover device equipped with a Vantec 500 detector.

Supplementary Information 1: IONC and PtNC recrystallization

Iron oxide nanocubes (IONCs) were synthesized according to the two-step synthesis described in the Materials and Methods section to obtain 13.3 ± 1.1 nm sized particles as shown in Figure SI1 a. Particle size evaluation has been performed by detecting the minimum diameter of detected particles within the TEM images via the OLYMPUS iTEM software and statistical analysis with Origin 2018. The average particle size was determined via a Gaussian fit as indicated by the red lines in Figure SI1.

In order to lower the average particle sizes from 13 to 12 nm, the heating rate during the synthesis of the particles has been increased to 3.5 °C/min and the duration at reflux temperature before cooling has been shortened to 20 minutes. The resulting particles are shown in Figure SI1 b and have an average size of 11.9 ± 1.0 nm. Recrystallization of the particle dispersions via the gas-phase diffusion technique allowed to further purify the particles by narrowing their size distribution and removing larger agglomerates. This purification process is illustrated in Figure SI1 c and d, as the obtained mesocrystals from each sample have a much smoother surface. The reduced size distribution is also indicated by the comparison of the Gaussian FWHM of both samples (before and after recrystallization) in Figure SI1 b and e. The average size of the recrystallized IONCs which were used for all further experiments was determined to be 11.9 ± 0.8 nm with a median aspect ratio (diameter max/diameter min) of 1.2 ± 0.1 via software-based evaluation of the TEM images.

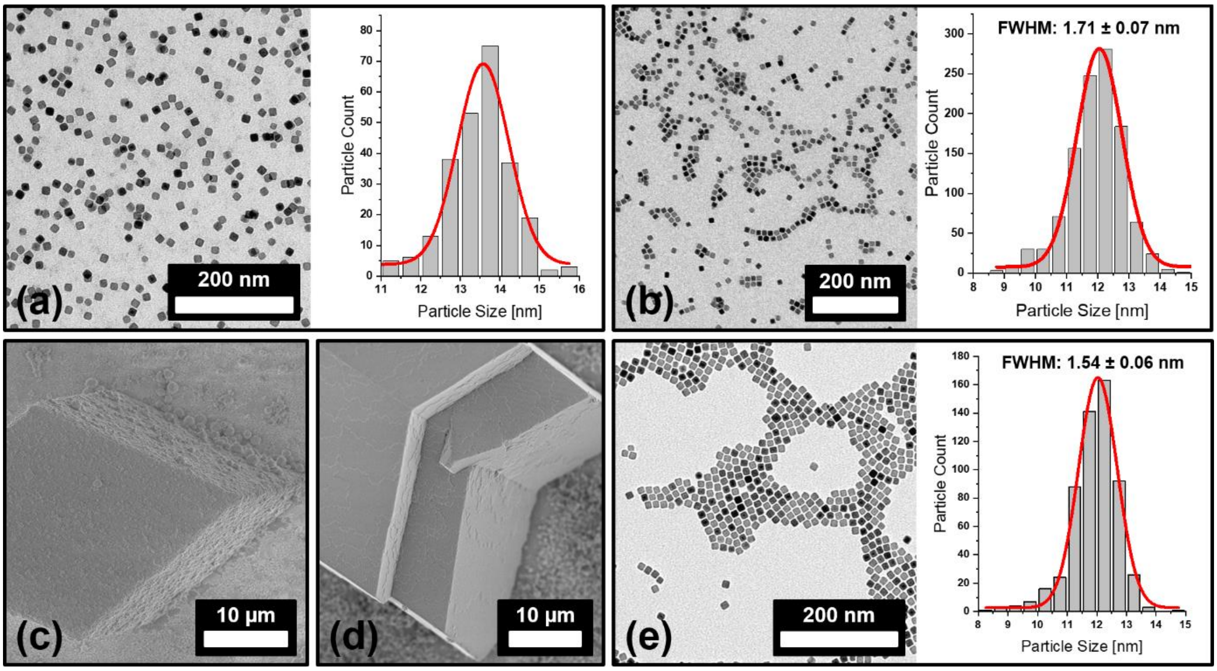

Figure SI1: TEM Images and their corresponding size distribution of iron oxide nanocubes determined by a particle detection software (Olympus iTEM) and their corresponding Gaussian fit in red (a, b and e). Comparison between (a) and (b) shows the particle size before (13.3 ± 1.1 nm) and after (11.9 ± 1.0 nm) changing the reaction parameters. Comparison between b and e illustrates the particle size distribution change after recrystallization (FWHM: 1.7 ± 0.1 nm to 1.5 ± 0.1 nm). (a) and (b) displays FE-SEM images of IONC mesocrystals formed from (a) crude (c) and recrystallized (d) particle dispersion.

Supplementary Information 2: HR-TEM analysis of PtNCs

High-Resolution TEM images at 1,000,000 times magnification (Figure SI2) reveal the atomic periodicity of individual nanoparticles, allowing the exact verification of the material due to their characteristic lattice plane distances. For pure platinum crystals the distances of the atomic planes have been determined to be 1.37 Å and 1.96 Å via Gatan Digital Micrograph Software which corresponds to the reported lattice planes of Pt[202] = 1.383 Å and Pt[200] = 1.956 Å according to the COD Entry_96-901-3418.

For binary particle mixtures, this method can be used in combination with FFT of the particles to quickly determine if the investigated particle is a PtNC or a IONC due to their characteristic atomic planes as shown in Figure SI2 c and d.

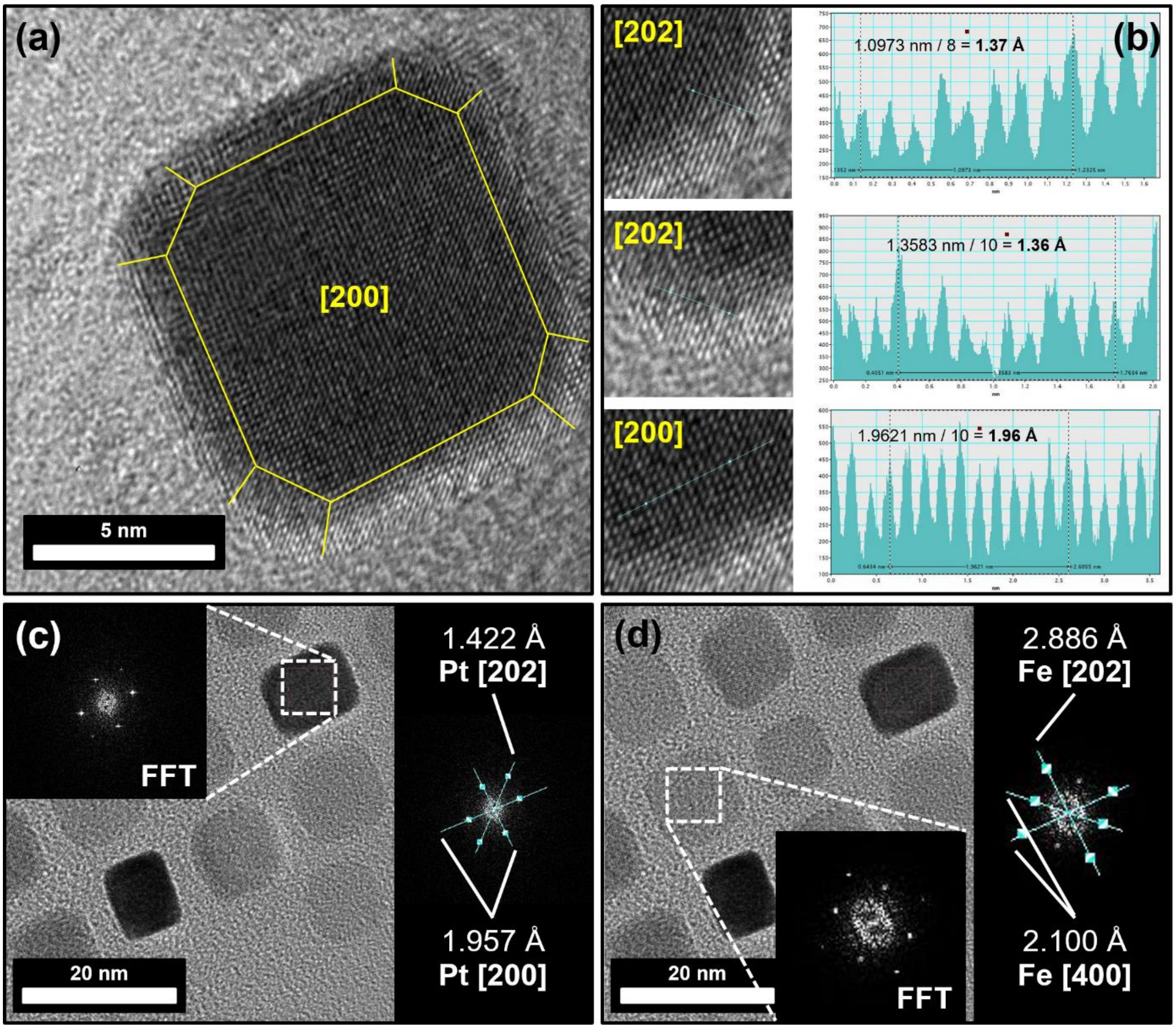

Figure SI2: HR-TEM images of a pure PtNC (a and b) and a binary particle mixture of PtNC and IONC (c and d). (b) illustrates the Gatan Digital Micrograph supported evaluation of the particle lattice planes via a pixel brightness line profile. In image (c) and (d), the periodicity of each particle has been determined by FFT to distinguish between PtNC and IONC.

Supplementary Information 3: Statistical Analysis of Pt-/IONC ratio

Software assisted (Olympus iTEM) particle detection allowed for a statistical analysis of binary PtNC and IONC self-assemblies to determine the ratio at which PtNCs are incorporated into a IONC host lattices. Both assemblies were prepared from 100 µL of a 1 mg/mL PtNC dispersion in hexane and 200 µL of a 10 mg/ml IONC dispersion in hexane resulting in a 1:20 wet mass ratio. The binary monolayers were prepared on a TEM grid (Figure SI3b and SI3c) and four large assemblies were counted by software to a total of 5669 particles of which particles below 30 nm² and above 200 nm²are excluded as they do not represent nanocubes participating in the self-assemblies leaving 5560 detected particles. According to HR-TEM images in Supplementary Information 2 we concluded that particles below an average brightness threshold above 200 (per software value) are platinum in the vast majority of the cases due to their significantly higher electron absorption of heavy and dense metals. The software determined 242 particles within the assemblies which inhere this low brightness and are therefore counted as PtNC. Figure SI3 (a) shows a detailed size distribution of the detected particles, where PtNCs are notably smaller within the assemblies when compared to the detected IONCs. This evaluation therefore gives a 4.35% ratio of PtNC within the solvent evaporation prepared 2D binary self-assemblies.

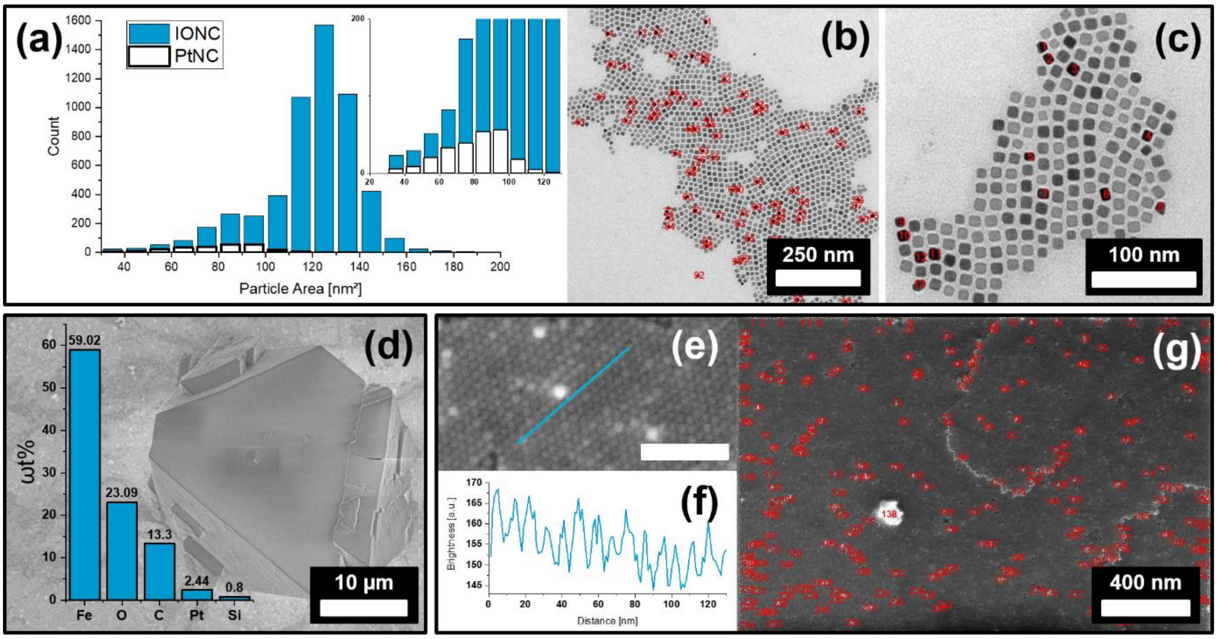

Figure SI3: Chart (a) shows the combined size distribution of IONCs and PtNCs by particle area [nm²] over 5560 software detected particles (Olympus iTEM) within four TEM images from which two examples are shown in (b) and (c). The inset in (a) displays a more detailed distribution of the PtNC. FE-SEM image (d) of a 3d binary PtNC/IONC mesocrystal illustrates how EDX analysis was performed to obtain elemental analysis of the mesocrystal (inset). The FE-SEM images (e) and (g) were performed on a broken inner surface of such a binary mesocrystal and used for determination of particle distances (e and f) as well as software assisted (Olympus iTEM) detection of high contrast platinum particles (g).

A similar statistical analysis has been performed on the inner surface of a broken 3D binary mesocrystal. Here, the brightness threshold for PtNC could not be crosschecked by the means of HR-TEM FFT analysis, however the significant brightness difference of the particles allowed for a reliable detection of these particles for RGB values above 100 based on the color profile of the image as shown in Figure SI3g. To reference the detected PtNC to the total amount of particles within the image, we measured the average particle distance on the investigated

surface to be 9.1 nm through a brightness profile as illustrated in Figure SI3e and SI3f. 235 detected PtNCs are therefore referenced to a total of 38774 particles, giving a 0.61% PtNC/IONC ratio for the inner mesocrystal surface. As the accuracy of this method can vary significantly based on the quality of the FE-SEM image, we verified our results by performing EDX analysis on three different crystals to determine the amount of various elements within the crystal. With an acceleration voltage of 10k eV the penetration depth of the electron beam will be several micro meters and therefore give a quite accurate representation of the mesocrystals bulk composition rather than its surface. Figure SI3d shows an example of such a measurement with the obtained wt% for the detected elements. The low amount of Si signals detected further assures that the electron beam does not penetrate the whole mesocrystal. Platinum was detected with a 2.44 wt% in this example and has to be accounted for its higher density before it is referenced to Fe_3O_4. Evaluation of all three measurements gives a median ratio of 0.68% with a small error of ± 0.04%, which is in good agreement with the 0.61% ratio for the software assisted particle detection method and further assures the feasibility of our results. However, it has to be stated that several presumptions, such as a unified particle size and equal distribution within the crystals, have to be made for the calculations with this method. Although the obtained values for the PtNC/IONC ratio with this method are therefore not highly accurate, they still give a good estimate on the amount of PtNCs that have been incorporated within the IONC mesocrystal host lattice.

Supplementary Information 4: Crystallographic evaluation of the binary mesocrystals SAXS pattern

The evaluation of the obtained SAXS pattern of our PtNC/IONC binary mesocrystals from the synchrotron measurements at the cSAXS beamline at the Paul Scherrer Institute suggests a tetragonal symmetry of the particle ordering. In combination with the radial integrated scattering curves a base centred tetragonal (BCT) packing of the particles was presumed. This presumption is backed by previous finding in our group which describe a tetragonal packing behaviour of self-assembled IONC due to the “bump-to-hollow” effect.[2] In combination with the determined predominant particle distances of 12.12 nm and 11.12 nm, the cell parameters can be calculated to be a = b = 34.24 nm and c = 29.24 nm for a unit cell suitable for a I4/mmm space group. The so constructed unit cell was simulated by using the CrystTBox software.[3] in order to find a corresponding zone axis which reflects the received synchrotron SAXS pattern and verifies our assumptions of a BCT unit cell. We found, that the theoretical scattering pattern for a [111] zone axis matches perfectly for the constructed unit cell and describes the majority of the measured reflexes as illustrated by the overlay in Figure SI4 d.

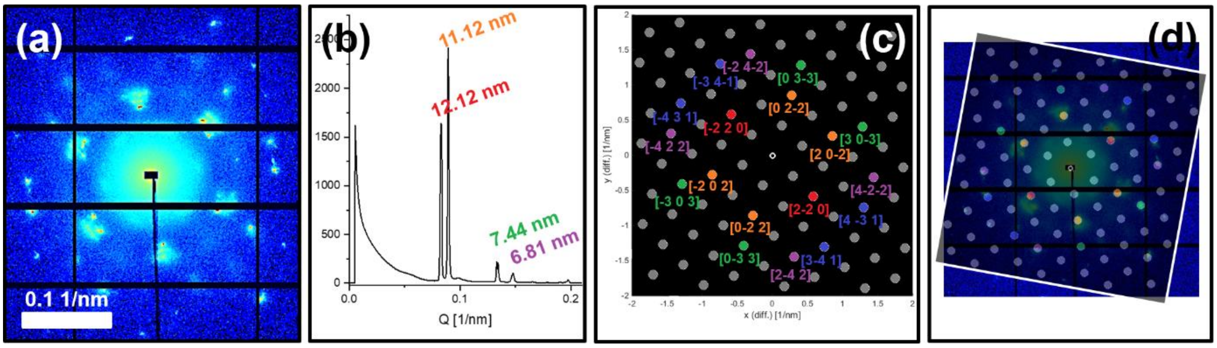

Figure SI4: Image (a) shows the synchrotron SAXS pattern of a PtNC/IONC binary mesocrystal and its corresponding radially integrated scattering signals in image (b). Image (c) illustrates the simulated diffraction pattern along the [111] zone axis of a I4/mmm space group unit cell with the parameters a = b = 34.24 nm and c = 29.24 nm. In colors are the reflexes highlighted matching with the measured SAXS pattern. Image (d) further displays this match with an overlay of the simulated pattern onto the actual SAXS pattern.

Supplementary Information 5: Analysis of Pt/Fe Mesocystals via SAXS

Experimental apparatus

Scanning SAXS was performed at the cSAXS beamline at the Paul Scherrer Institute with a synchrotron-based x-ray beam focused to 27 x 16 µm and monochromatized by a fixed-exit double Si(111) monochromators at an energy of 11.2 keV. 2D scattering signal was recorded on a Pilatus 2M detector placed at 2.1928 m from the measured sample calibrated from scattering pattern of silver behenate.[4] The transmitted beam intensity was measured with a photodiode placed on the surface of a beamstop inside the steel flight tube under vacuum conditions.

A dispersion of the mesocrystals on kapton tape was done prior to the beamtime and measured in scanning mode with an exposure time of 0.8 seconds per points with a step size of 20 µm x 20 µm.

Integration of 2D scattering images and radiation damage check was performed following the azimuthal segment procedure using the cSAXS Matlab analysis package.[5]

Determination of inter-particle gap

The complex scattering of the recorded mesocrystals displayed a combination of diffraction peaks associated to the tetragonal packing of the cubic nanoparticles as well as the form factor of the single particles superposed with a peak at ~0.58 nm^{-1}, which we assume to be the inter-particle size – the average length between the mass centres of two nanoparticles in the tetragonal packing. In order to estimate the average inter-particle gap – as the size of the two half particles minus the inter-particle distance, we have isolated the signature from the cubic form factor first by analysing the segmented radially integrated scans.[5] The full detector image was radially segmented into 16 azimuthal segments and each of them individually integrated. Segments without peak at ~0.58 nm^{-1} were selected and averaged in order to improve the signal to noise ratio (Figure SI5.1 - left). Similar procedure was performed for segments showing a peak at ~0.58 nm^{-1} (Figure SI5.1 - right).

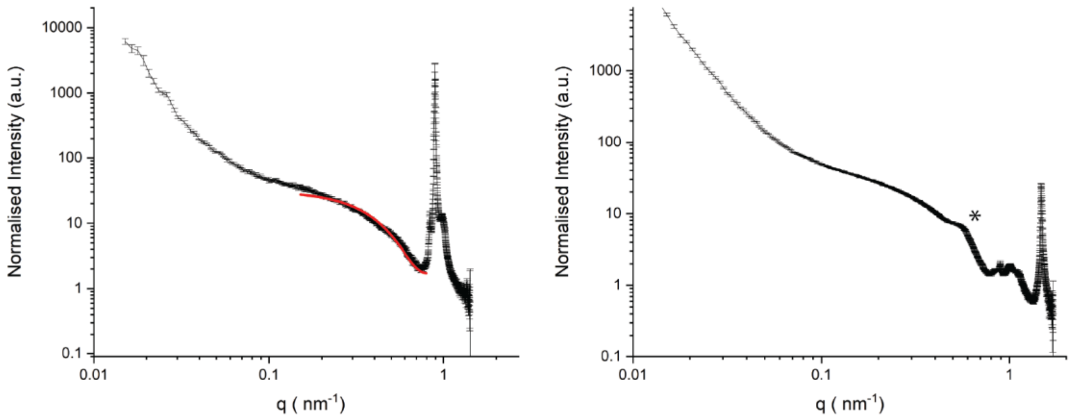

Figure SI5.1: Radially integrated segments presenting without the inter-particle peak (left) with best fit of cubic nanoparticles of 8.8±0.8 nm^{-1} (red curve) and radially integrated segments (right) showing the inter-particle peak (*).

The form factor was estimated using SASView 5.0.04 package and a rectangle prism model where for consideration of a cubic shape, the side ratios length was fixed at 1. In order to avoid the contribution of a structure factor, the fitted q-range was selected between 0.15

and 0.8 nm^{-1}. The cube length from the fitted scattering curve gave an estimated size of 8.8 ± 0.8 nm. It has to be noted that this size is slightly lower than the estimated one from TEM in this study but arises from several factors. Firstly, estimation of the particle size from the TEM analysis might be slightly overestimated if the particles do not lay perfectly flat or the imaging angle is different from 90°. Taking this aspect into account, the size of the cubic particles as determined via SAXS as a = 8.8 nm would possess a diagonal cube length in the same order of size as defined via TEM as per:

$$Diagonal\ cube\ length = \sqrt{2 * a^2} = 12.4\ nm$$

Secondly, the fitted value is a single measurement averaging several hundreds of nanoparticles. Ultimately, the determination of a form factor is usually established for dilute systems and becomes complicated for such densely packed systems.

The latter also complicates the separation of structure and form factor. Therefore, we follow a simplified approach to estimate the inter-particle distance – the average length between the mass centres of two nanoparticles in the tetragonal packing – from the scattering curves. We treated the fitted profiles, corresponding to the cubic form factor of the individual nanoparticles as a background and subtracted them from the averaged and integrated segments of the detector image where the particle peak at ~0.58 nm^{-1} was observed. The residual was fitted with a Gaussian curve to determine the peak centre and FWHM. The average length between the mass centres of two nanoparticles in the tetragonal packing – as per fitted curves – was calculated to be 11.12±1.30 nm. The inter-particle gap gives an average value over all directions of the tetragonal packing of 2.54±1.56 nm (Figure SI5.2).

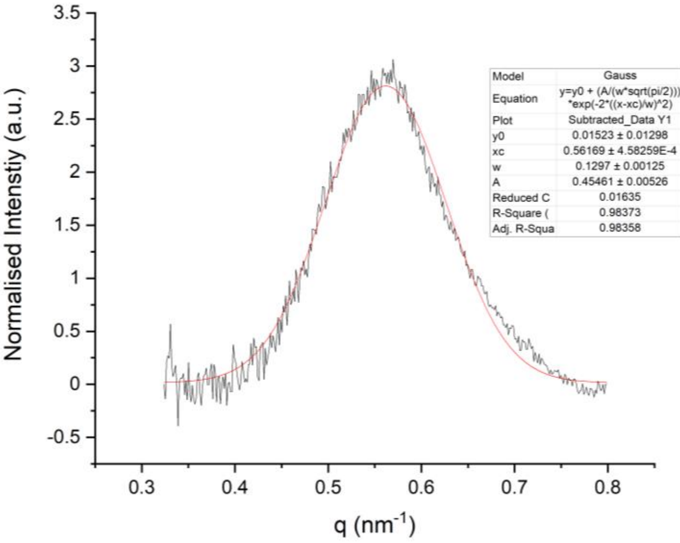

Figure SI5.2: Best Gaussian fit for inter-particle distance peak obtained from the subtraction of the cubic modelling fit to the radially integrated scattering curves between 0.32 and 0.8 nm^{-1}.

References

[1] *J. Am. Chem. Soc.* **2009**, *131*, 18543–18547, *doi:10.1021/ja908245r.*

[2] *Adv. Mater. Interfaces* **2017**, *4*, 1600431

[3] M. Klinger. CrysTBox - Crystallographic Toolbox. Institute of Physics of the Czech Academy of Sciences, 2015. URL: http://www.fzu.cz/~klinger/crystbox.pdf.
[4] P. Kraft, A. Bergamaschi, C. Broennimann, R. Dinapoli, E. F. Eikenberry, B. Henrich, I. Johnson, A. Mozzanica, C. M. Schleputz, P. R. Willmott, B. Schmitt, *J. Synchrotron Radiat.* **2009**, *16*, 368.
[5] Scanning SAXS Software Package, https://www.psi.ch/en/sls/ csaxs/software (accessed: April 2021).
